# Collateral Arteriogenesis Involves a Sympathetic Denervation That Is Associated With Abnormal α-Adrenergic Signaling and a Transient Loss of Vascular Tone

**DOI:** 10.3389/fcvm.2022.805810

**Published:** 2022-02-15

**Authors:** Alexander Silva, Christopher J. Hatch, Megan T. Chu, Trevor R. Cardinal

**Affiliations:** Biomedical Engineering, California Polytechnic State University, San Luis Obispo, CA, United States

**Keywords:** arteriogenesis, vasodilation, vascular tone, sympathetic, adrenergic, ischemia

## Abstract

Stimulating collateral arteriogenesis is an attractive therapeutic target for peripheral artery disease (PAD). However, the potency of arteriogenesis-stimulation in animal models has not been matched with efficacy in clinical trials. This may be because the presence of enlarged collaterals is not sufficient to relieve symptoms of PAD, suggesting that collateral function is also important. Specifically, collaterals are the primary site of vascular resistance following arterial occlusion, and impaired collateral vasodilation could impact downstream tissue perfusion and limb function. Therefore, we evaluated the effects of arteriogenesis on collateral vascular reactivity. Following femoral artery ligation in the mouse hindlimb, collateral functional vasodilation was impaired at day 7 (17 ± 3 vs. 60 ± 8%) but restored by day 28. This impairment was due to a high resting diameter (73 ± 4 μm at rest vs. 84 ± 3 μm dilated), which does not appear to be a beneficial effect of arteriogenesis because increasing tissue metabolic demand through voluntary exercise decreased resting diameter and restored vascular reactivity at day 7. The high diameter in sedentary animals was not due to sustained NO-dependent vasodilation or defective myogenic constriction, as there were no differences between the enlarged and native collaterals in response to eNOS inhibition with L-NAME or L-type calcium channel inhibition with nifedipine, respectively. Surprisingly, in the context of reduced vascular tone, vasoconstriction in response to the α-adrenergic agonist norepinephrine was enhanced in the enlarged collateral (−62 ± 2 vs. −37 ± 2%) while vasodilation in response to the α-adrenergic antagonist prazosin was reduced (6 ± 4% vs. 22 ± 16%), indicating a lack of α-adrenergic receptor activation by endogenous norepinephrine and suggesting a denervation of the neuroeffector junction. Staining for tyrosine hydroxylase demonstrated sympathetic denervation, with neurons occupying less area and located further from the enlarged collateral at day 7. Inversely, MMP2 presence surrounding the enlarged collateral was greater at day 7, suggesting that denervation may be related to extracellular matrix degradation during arteriogenesis. Further investigation on vascular wall maturation and the functionality of enlarged collaterals holds promise for identifying novel therapeutic targets to enhance arteriogenesis in patients with PAD.

## Introduction

Peripheral artery disease (PAD) affects ~8.5 million people in the United States and most frequently manifests as intermittent claudication, or pain while walking (1, 2). PAD is an ischemic disease typically caused by atherosclerosis of the lower extremities, and the most severe form of PAD is critical limb ischemia (CLI), which affects roughly 11% of patients (3). CLI occurs when severe blockage in the arteries of the lower extremities cause rest pain, gangrene, and chronic ulcerations (4). Approximately 20–30% of the individuals with CLI cannot undergo percutaneous intervention or bypass surgery due to comorbidities (5, 6), resulting in a 20% 5-year mortality rate (7). Alternatively, enlargement of natural bypass collaterals through arteriogenesis generally improves patient prognosis with coronary ischemic disease (8–10), and the presence of collaterals is associated with improved walking performance in patients with PAD (11). These observations highlight the stimulation of collateral arteriogenesis as an attractive therapeutic target. Unfortunately, despite success in animal models (12–14) and unrandomized or unblinded clinical trials (15), delivering growth factors involved in arteriogenesis or transplanting bone-marrow derived cells have failed to enhance collateral arteriogenesis or improve functional outcomes in numerous double-blinded, placebo controlled, randomized clinical trials (15–17). This failure, coupled with the observation that collaterals are not always protective, suggests that the benefit of collaterals may depend on more than their mere presence. Specifically, larger and more numerous collaterals can be associated with reduced limb perfusion and greater prevalence of symptomatic PAD (18), and half of patients with enlarged coronary collaterals still experience ischemic pain during physical activity (19, 20).

An alternative explanation is that symptomatic PAD is due to, or at least exacerbated by, impaired vasodilation in enlarged collaterals (21). Vasodilation is impaired in both the upper (22) and lower (23) extremities in patients with PAD. Furthermore, vasoconstrictor administration for the treatment of headaches aggravates claudication symptoms (24, 25). These observations suggest that a reduced ability to increase blood flow contributes to symptomatic disease presentation, which is supported by the inverse correlation between disease severity and exercise blood flow/flow reserve to the ischemic zone (26). From a hemodynamic perspective, blood flow control to the ischemic zone could be impaired due to abnormal vascular reactivity in arterioles and small arteries of the ischemic skeletal muscle or in upstream collaterals. Because the collateral circulation is the primary site of vascular resistance following arterial occlusion (27–29), impaired vasodilation of collaterals would have the greatest impact on downstream tissue perfusion. Therefore, it might not be sufficient for collaterals to re-perfuse downstream tissue solely by acting as larger conductance pathways. Collaterals should also be able to regulate blood flow to match oxygen delivery with tissue metabolic demand (30), and abnormal vascular reactivity might limit the therapeutic effects of collaterals (31). Normally, blood flow to individual muscles is controlled locally to ensure the matching of blood flow with tissue metabolic demand (32). However, following ischemia, blood flow is controlled by a high resistance collateral circulation that must have a functional range to accommodate varying levels of tissue metabolic demand throughout the entire lower limb (33). Interestingly, most investigators evaluate collaterals by measuring maximum diameter (34–36), but this does not provide information about resting diameter and vascular reactivity, which defines the ability of a resistance vessel to regulate blood flow. To identify new therapeutic targets for patients with PAD, a greater understanding of vascular reactivity following vessel adaption is needed (37).

While patients with PAD exhibit reduced vascular reactivity, there is no consensus as to the underlying mechanisms in humans (38, 39), and surprisingly little is known about vascular reactivity of collaterals in laboratory animals to identify potential therapeutic targets. Collateral-dependent blood flow is impaired in rodents (40–42), as it is in humans, but regional blood flow measurements are unable to distinguish between collateral diameter, collateral number, and collateral reactivity. In the ischemic or collateral-perfused zone, rodents, like humans, exhibit impaired endothelial-dependent vasodilation in intramuscular arterioles (43). This fits with the hypothesis that endothelial dysfunction causes impaired vasodilation in patients with PAD (44). However, patients with PAD have significantly higher plasma NO levels compared to non-PAD patients (45), suggesting that the lack of vasodilation may be caused by insensitivity of SMCs to NO, not an inability of ECs to produce NO (i.e., endothelial dysfunction) (38). While vasodilation in rodent collaterals is impaired in response to endothelial-dependent agents (31, 46) these observations were made without concomitant evaluation of smooth muscle-dependent vasodilation. Therefore, apparent endothelial dysfunction in collaterals could be due to smooth muscle dysfunction, as it is in feed arteries of the ischemic zone (47).

The closest comprehensive observation of vascular reactivity in collaterals *in vivo* comes from arterialized collateral capillaries (48). Collateral capillaries anastomose terminal arterioles of adjacent arteriolar trees, and can undergo arterialization into functional collaterals when the feed artery of one of the arteriolar trees is obstructed (49). In arterialized collateral capillaries, vasodilation is largely absent at day 7 following arterial occlusion, but is restored and capable of increasing perfusion to the ischemic zone by day 21 (48). Interestingly, impaired vasodilation in arterialized collateral capillaries, like that of feed arteries in the ischemic zone (47), is due to impaired smooth muscle cell function. This is not surprising, given that in an arterialized collateral capillary, a non-reactive capillary becomes a reactive collateral arteriole following the recruitment or transdifferentiation of vascular smooth muscle cells (50). A proinflammatory environment, such as that during collateral capillary arteriogenesis, would be expected to cause vascular smooth muscle cells to exhibit a synthetic, largely non-contractile phenotype (51, 52) with reduced contractile signaling (53).

In the case of native collaterals, enlargement occurs from pre-existing arteriole-arteriole anastomoses. While vascular smooth muscle cells in native collaterals also transition from a contractile to a proliferative and synthetic phenotype during arteriogenesis (51), they already reside in the medial layer of arterioles and do not need to be recruited from surrounding tissue or neighboring vessels. Therefore, it is unclear if vascular smooth muscle cells would drive the vasodilatory phenotype of enlarging native collaterals to the same extent as in enlarging arterialized collateral capillaries. To determine this, we tested the hypothesis that vasodilation is impaired in enlarged collateral arterioles of the mouse hindlimb following femoral artery ligation and set out to evaluate the mechanism of any impairment observed.

## Materials and Methods

### Animals

All procedures were performed according to protocols approved by the California Polytechnic State University Institutional Animal Care and Use Committee. Male and female C57Bl/6 mice (Taconic Farms or Jackson Labs) between 2 and 4 months of age were used for all experiments. For all studies, a single replicate indicates a single collateral, from the sham operated or ligated gracilis anterior muscle, from a single mouse. All mice were maintained on a 12-h light/dark cycle and given feed and water ad libitum. Mice were housed in microisolator cages with enrichment (nesting material, “house,” and plastic tube). Access to an exercise wheel (Columbus Instruments 8 Station Home Cage Running Wheel System) was provided post-surgery for mice in the voluntary exercise study.

### Femoral Artery Ligation

Chronic hindlimb ischemia was induced by ligating the femoral artery between the epigastric and popliteal branches, as described previously (54). Briefly, animals were anesthetized in an induction chamber with 5% isofluorane in oxygen flowing at 0.8–1.0L·min^−1^. Following induction, isofluorane was decreased to 2–3%. Hair was removed from the ventral aspect of both hindlimbs using depilatory cream, and skin was disinfected using chlorhexidine diacetate. Veterinary ophthalmic ointment was placed over the eyes to prevent corneal desiccation, and mice were given pre-operative buprenorphine analgesic (0.075 mg·kg^−1^, subcutaneous). Body temperature was maintained at 35°C with a temperature-controlled heat pad and a rectal thermistor probe (CWE, Inc.; Ardmore, PA). The left femoral artery was separated from the paired nerve and vein, and ligated with 6-0 silk suture; the skin incision was closed with 7-0 polypropylene suture. A sham surgery was performed in the right hindlimb, in which the connective tissue overlying the femoral neurovascular bundle was gently blunt dissected before skin closure. Mice were given post-operative buprenorphine analgesic and allowed to recover on a heat pad until ambulatory; post-operative buprenorphine analgesic was continued for 2–3 days following surgery.

### Functional Vasodilation

At day 7 or 28 following femoral artery ligation, animals were anesthetized and prepared as described above. The anterior gracilis muscle was exposed via skin incision and resection of the epigastric fat pad; overlying fascia was removed by gentle blunt dissection. A custom trans-illuminating light emitting diode (LED) was placed under the hindlimb to allow visualization of the gracilis anterior collateral, and two tungsten microelectrodes (FHC; Bowdoin, ME) were placed near the motor-end plates of the gracilis anterior, halfway between where the profunda femoris artery and the saphenous artery cross the muscle. A stimulus isolator and data acquisition system (ADInstruments; Colorado Springs, CO, USA) were used to stimulate muscle contractions with 1 mA square waves of 0.2 ms duration at 1 Hz to confirm proper electrode placement. The preparation was irrigated with PBS, covered with plastic wrap to prevent desiccation, and given 30 min to equilibrate. After equilibration, the gracilis muscles were stimulated for 90 s with 1 mA square waves of 0.2 ms duration at 8 Hz. Images of the collateral midzone were captured before and immediately after stimulation using an intravital microscope (Olympus BXFM) with a 10 X ultra long working distance objective (Olympus LMPlanFL N), CCD camera (Retiga 2000R), and digital imaging software (QCapture Pro). The entire procedure was repeated for the contralateral hindlimb.

### *In vivo* Vascular Reactivity

At day 7 or 28 following femoral artery ligation, animals were anesthetized and prepared as described above. The intravital microscope was placed over the gracilis anterior collateral and the trans-illuminating LED probe was placed under the hindlimb. A physiological salt solution (PSS) containing (in mM) NaCl 137, KCl 4.7, MgSO_4_ 1.2, CaCl_2_ 2, and NaHCO_3_ 18 was deoxygenated with 5% CO_2_-95% N_2_, maintained at ~35°C, and flowed over the exposed collateral at ~2 ml min^−1^ for an equilibration period of at least 30 min. Images were captured at rest and during the maximum diameter change within 5 min after application of a maximal dose (10^−4^ M) of each pharmacological agent: nitric oxide synthase inhibitor Nω-Nitro-L-arginine methyl ester hydrochloride (L-NAME, Sigma-Aldrich), α-adrenergic agonist norepinephrine (Sigma-Aldrich), L-type calcium channel blocker nifedipine (Cayman Chemical), and α-adrenergic antagonist prazosin (Cayman Chemical). If multiple agents were used in the same preparation, a 30 min “washout” period was observed between each agent. The entire procedure was then repeated for the contralateral hindlimb.

### Perfusion Fixation

At day 7 or 28 following femoral artery ligation, perfusion fixation was used to preserve the gracilis anterior muscles. Before perfusion, gracilis anterior muscles were separated from surrounding fascia to facilitate resection. After exposing the thoracic cavity, a 27G needle was inserted into the left ventricle via the apex of the heart, and the right atrium was cut for perfusate efflux. Next, a pre-warmed PBS solution containing 10^−3^ M sodium nitroprusside (vasodilator), 10^−4^ M adenosine (vasodilator), and 100 U·ml^−1^ heparin (anticoagulant) was perfused at 4 ml·min^−1^. Lastly, 5 ml of cold 4% paraformaldehyde was perfused through the left ventricle. Once fixed, gracilis anterior muscles were resected from the profunda femoris artery to the distal saphenous artery.

### Immunofluorescence

Fixed gracilis anterior muscles were frozen, embedded in tissue processing medium (OCT compound), and stored at −80°C until further use. Muscles were sectioned transversely at 20 μm, collected directly onto pre-treated tissue slides, and labeled with fluorescent antibodies to visualize arterioles (a-smooth muscle actin), sympathetic neurons (tyrosine hydroxylase), matrix metalloproteinase 2, and nuclei (bisbenzimide). Briefly, tissue sections were rinsed in PBS 3x for 5-min to remove OCT and permeabilized in 2% Triton-X-100 in PBS for 20 min at room temperature. Sections were then stained with 0.1% Triton-X-100, 2% BSA, 1:300 Anti-Alpha -Smooth Muscle Actin – Cy3 antibody (Sigma-Aldrich C6198), 1:100 Anti-Tyrosine Hydroxylase Antibody (rabbit, anti-mouse, Millipore Sigma, AB152), or 1:100 Anti-MMP2 (rabbit, anti-mouse, Proteintech, 10373-2-AP) in PBS. Slides were incubated at room temperature for 12 h in a humidified box. They were then washed with PBS 3 x for 10 min and incubated with 1:100 of secondary Goat anti-Rabbit IgG Cross-Adsorbed Secondary Antibody, Alexa Fluor 488 (ThermoFisher A11008) for 2 h at room temperature. Tissue sections were washed with PBS 3 x for 10 min and incubated with bisbenzimide (BBI) for 30 min at room temperature followed by washing with 0.1% Triton-X-100 in PBS 3 x for 10 min. The slides were placed in a final 30-min wash in PBS at room temperature prior to cover-slip application. Tissue sections were stored at 4°C until imaging. Control labeling was performed by omitting the primary or the secondary antibodies. Images of collateral vessels from tissue sections in the midzone (i.e., halfway between the profunda femoris and saphenous arteries) were captured with a confocal microscope (Olympus FV1000) at 10X and 40X using the DAPI (ex. 359 nm, em. 457 nm), Alexa Fluor 488 (ex. 490 nm, em. 525 nm), and Cy3 (ex. 554 nm, em. 568 nm) filter settings. Images at 40X were captured after identifying the region of interest around the main collateral vessel in each transverse cross section; 3 sections per muscle were measured.

### Statistics

Data are presented as mean ± SEM. Statistical significance was set at *p* ≤ 0.05. Vessel diameters were measured with Image/J software and percent change from resting diameter was calculated; increases above 0% represent vasodilation while decreases below 0% indicate vasoconstriction. For vessel diameter measurements, image brightness was adjusted to improve contrast and collaterals without clear luminal borders were excluded. Differences between resting and treated diameter were determined by paired *t-*test with a Bonferroni correction. Differences between resting diameter, treated diameter, or percent change in diameter between ligated and sham-operated limbs and between day 7 and day 28 time points were determined using two-wav ANOVA followed by Tukey *post-hoc* tests. Tyrosine Hydroxylase and MMP-2 density was expressed as percentage of area occupied by threshold signal divided by the total area of interest; the threshold signal was determined based on primary antibody-only and secondary antibody-only negative control sections. Distance measurements between fluorescence signals for sympathetic innervation (TyH) surrounding the primary collateral vessels in the region of interest were determined with Image/J. The distance between collateral vessels (red) and TyH signal (green) was measured using straight line tool, with numerous measurements in the region of interest; the closest distance between the transverse collateral vessel and the detected signal was selected. Differences in TyH and MMP2 staining between sham-operated and ligated limbs and between day 7 and 28 time points were determined using two-way ANOVA followed by Tukey *post-hoc* tests.

## Results

**Figure 1:**
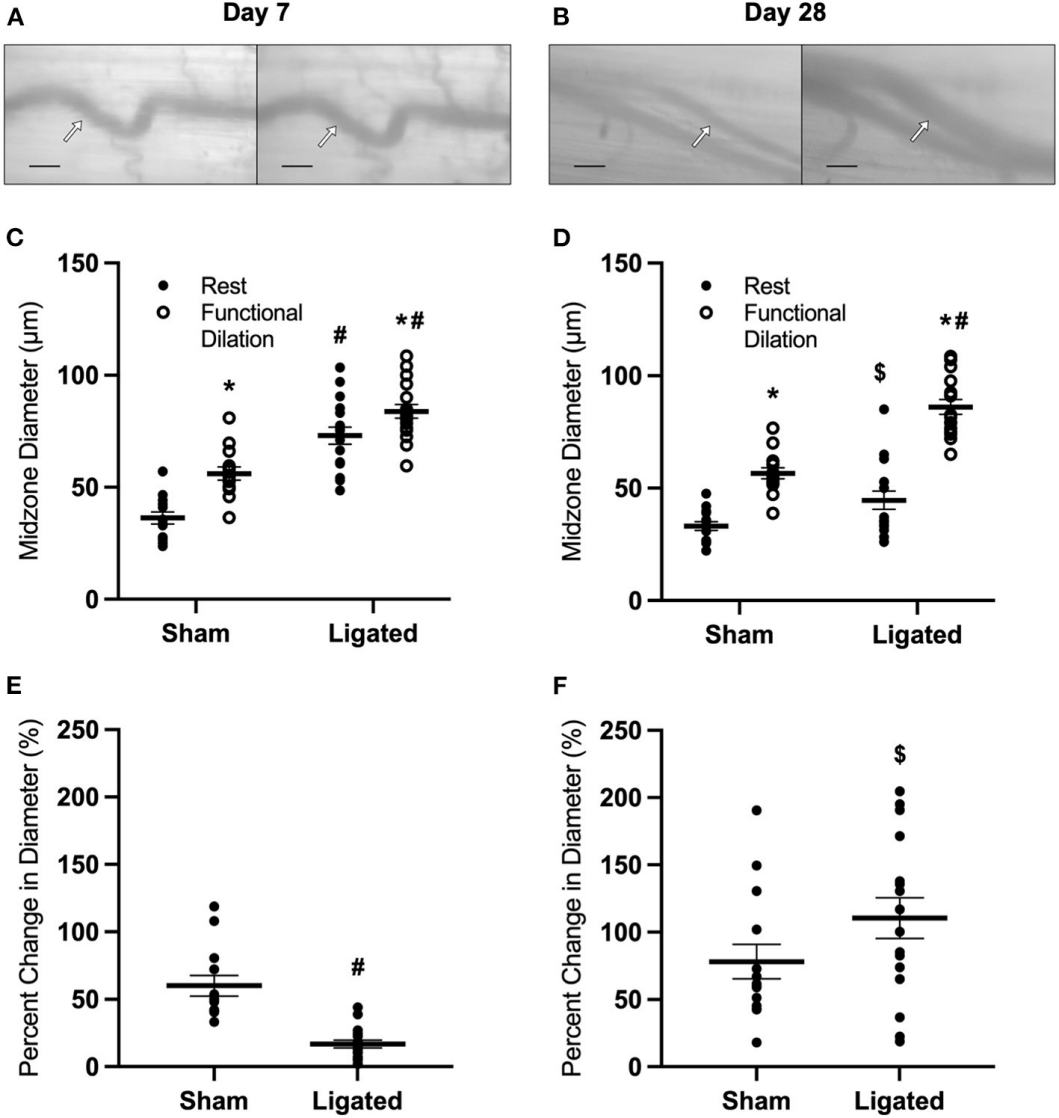
Collateral functional vasodilation is impaired at day 7 and restored at day 28. Collateral vessels were observed at the collateral midzone by intravital microscopy with transillumination at 7 days **(A)** or 28 days **(B)** post-femoral artery ligation in the left mouse hindlimb at rest (left) and following functional vasodilation (right); arrow indicates the enlarged collateral arteriole and scale bar represents 100 μm. At each time point, the gracilis muscles of C57Bl/6 mice (*n* = 17, 9 male and 8 female, at day 7 and *n* = 16, 8 male and 8 female, at day 28) were stimulated with tungsten microelectrodes and a stimulus isolator. At day 7, both resting and maximum diameters were increased in the ligated limb compared to the sham **(C)**, and vascular reactivity was decreased **(E)**. At day 28, maximum diameters remained increased in the enlarged collateral in comparison to the native collateral, but resting diameter was smaller than at day 7 **(D)**. Vascular reactivity in the enlarged collateral at day 28 was greater than at day 7, and was no longer different from the native collateral **(F)**. *p < 0.05 vs. resting, #*p* < 0.05 vs. sham, $ *p* < 0.05 vs. day 7.

To test the hypothesis that vasodilation is impaired following femoral artery ligation, we used intravital microscopy to assess functional vasodilation in the collateral midzone at 7 or 28 days following ligation (Figures 1A,B). By day 7, the collateral arterioles had enlarged by arteriogenesis, as evidenced by the increased maximum diameter in the enlarged collateral (84 ± 3 vs. 56 ± 3 μm, Figure 1C). Interestingly, resting diameter in the enlarged collateral (73 ± 4 vs. 36 ± 3 μm in the native collateral) was very close to the maximum diameter. This indicates that arteriogenesis reduced vascular reactivity, i.e., the difference between resting and dilated diameters, with a percent increase in diameter of only 17 ± 3% in the enlarged collateral vs. 60 ± 8% in the native collateral (Figure 1E). As expected, structural enlargement induced by arteriogenesis was maintained through day 28, as demonstrated by larger maximum diameter in the enlarged collateral as compared to the native collateral (86 ± 3 vs. 57 ± 2 μm, Figure 1D). However, resting diameter in the enlarged collateral was smaller at day 28 (45 ± 4 μm) than at day 7, and vascular reactivity was restored, as the percent increase in diameter was not different between enlarged and native collaterals (110 ± 15 vs. 78 ± 13%, Figure 1F).

**Figure 2:**
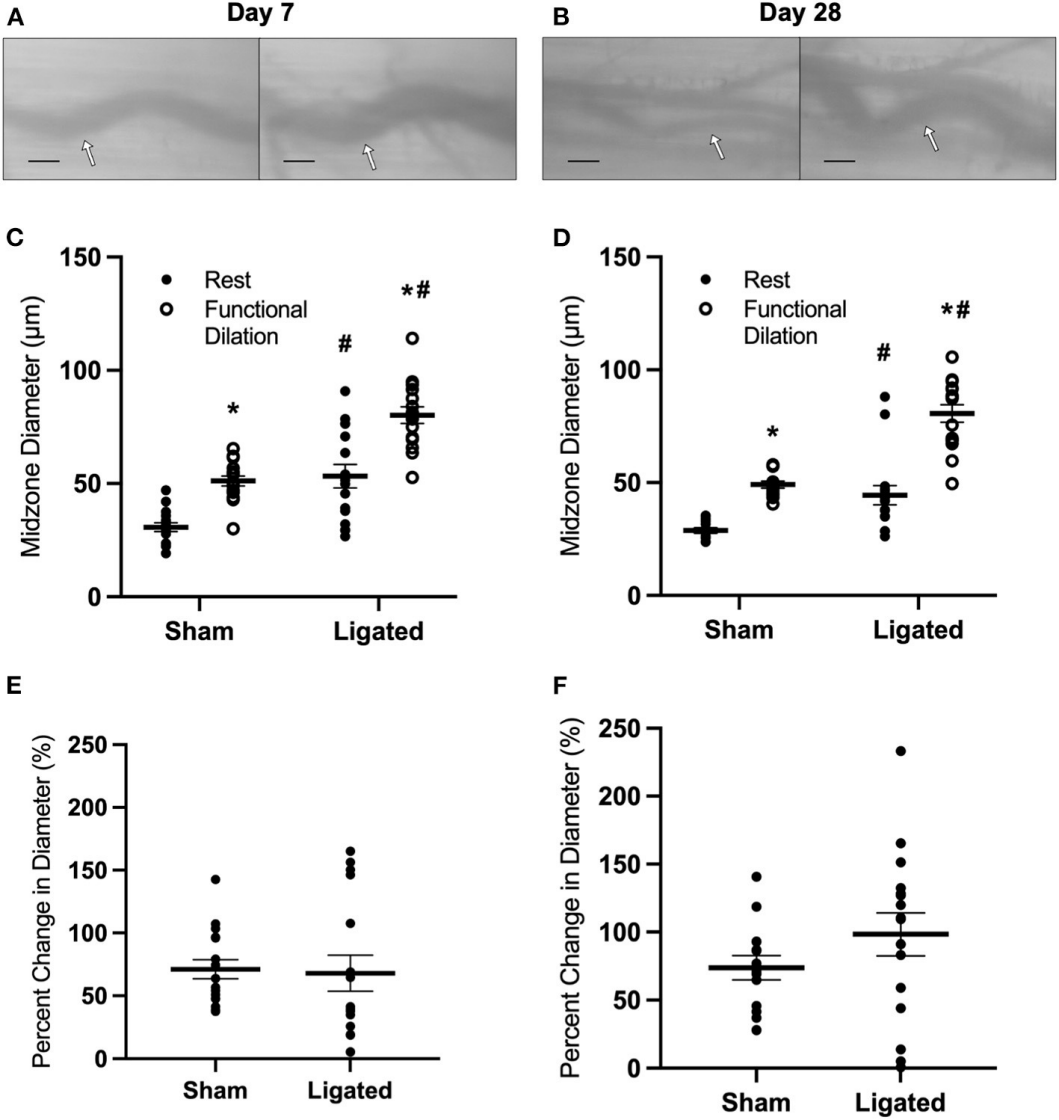
Collateral functional vasodilation is improved with voluntary exercise. Collateral vessels were observed at the collateral midzone by intravital microscopy with transillumination at 7 days **(A)** or 28 days **(B)** post-femoral artery ligation in the left mouse hindlimb at rest (left) and following functional vasodilation (right); arrow indicates the enlarged collateral arteriole and scale bar represents 100 μm. At each time point, the gracilis muscles of C57Bl/6 mice (*n* = 16, 8 male and 8 female, each at day 7 and day 28) were stimulated with tungsten microelectrodes and a stimulus isolator. At day 7, resting diameter in the enlarged collateral was decreased in mice that performed voluntary exercise **(C)**, resulting in increased vascular reactivity; resting diameter was similarly low at day 28 **(D)**. Therefore, vascular reactivity was not different between enlarged and native collaterals of mice that performed voluntary exercise at both day 7 and day 28 **(E,F)**. *p < 0.05 vs. resting, #*p* < 0.05 vs. sham.

The large resting diameter at day 7 following ligation may allow for maximum blood flow to downstream hypoxic tissue, which could be a beneficial effect of collateral enlargement. If this were the case, then we would expect that increased downstream tissue metabolism would further increase resting diameter of the enlarged collateral at day 7 and/or delay the restoration of vascular reactivity. Therefore, we determined the impact of voluntary exercise on collateral diameter and functional vasodilation at 7 or 28 days following ligation (Figures 2A,B) by placing a running wheel in the home cage. Surprisingly, voluntary exercise decreased resting diameter in the enlarged collateral at day 7 (53 ± 5 μm, Figure 2C), such that there was no difference in vascular reactivity between enlarged and native collaterals at day 7 (68 ± 14 vs. 71 ± 8%, Figure 2E) nor at day 28 (98 ± 16 vs. 74 ± 9%, Figure 2F). These results indicate that a higher resting diameter is not a beneficial effect of collateral enlargement, and lead us to evaluate the mechanism of this increase.

**Figure 3:**
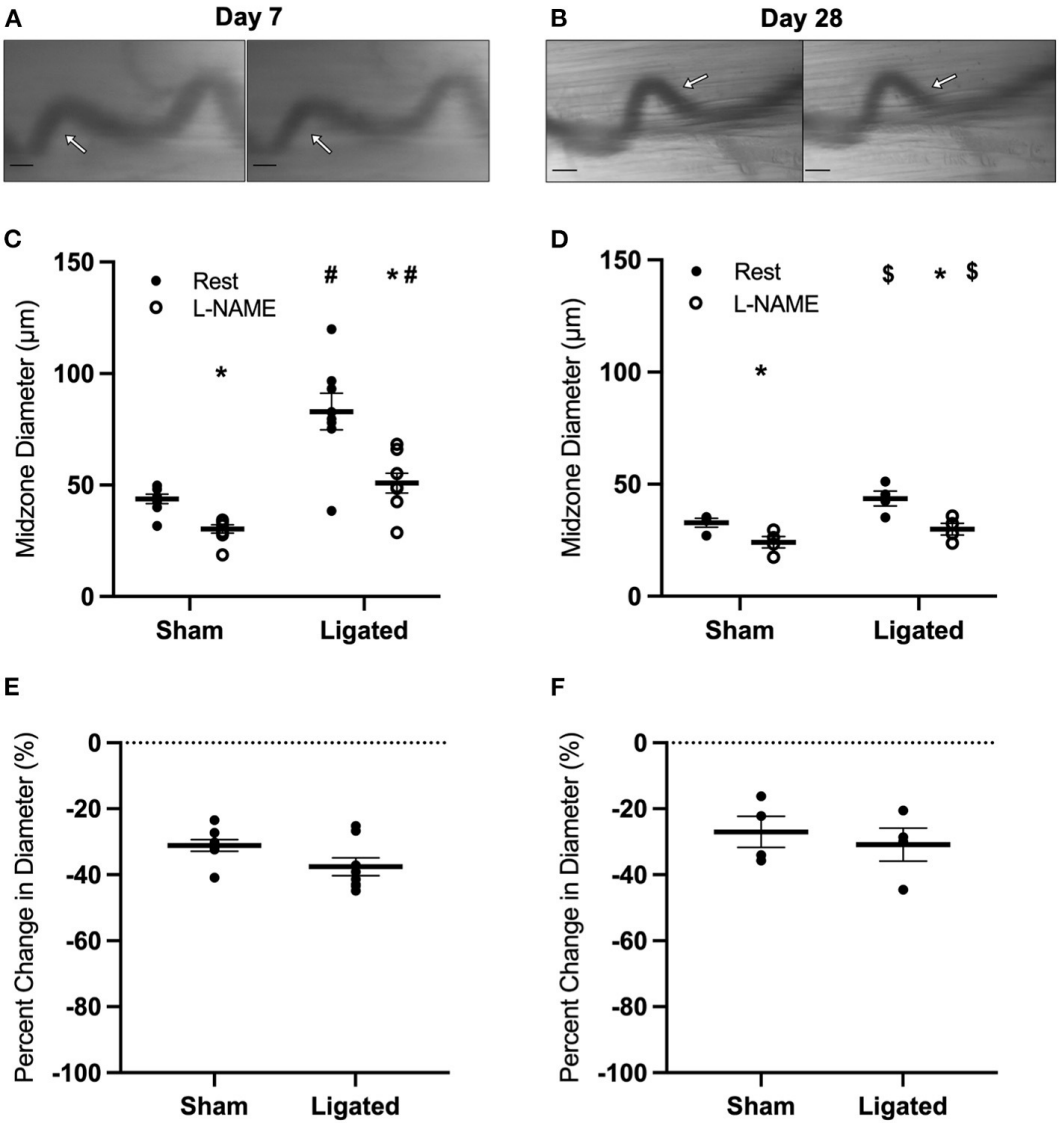
Collateral vessels are not more sensitive to nitric oxide inhibition at day 7 and day 28. Collateral vessels were observed at the collateral midzone by intravital microscopy with transillumination at 7 days **(A)** or 28 days **(B)** post-femoral artery ligation in the left mouse hindlimb at rest (left) and following exposure to 10^−4^ M of nitric oxide inhibitor L-NAME (right); arrows indicate the enlarged collateral arteriole and scale bar represents 100 μm. Nitric oxide inhibition vasoconstricts collaterals at both day 7 (**C**, *n* = 8) and day 28 (**D**, *n* = 4). However, there is no difference in the vascular reactivity between the native and enlarged collaterals at both 7 days **(E)** or 28 days **(F)** post-femoral artery ligation. *p < 0.05 vs. resting, #*p* < 0.05 vs. sham, $*p* < 0.05 vs. day 7.

Because arterial occlusion induces a shear-induced vasodilation immediately following ligation (55, 56), we next determined if the higher resting diameter at day 7 reflected a maintenance of the initial vasodilation induced by ligation. Because this initial vasodilation is due to endothelial nitric oxide (NO) production, we measured the response of the collateral mid-zone at 7 or 28 days following ligation (Figures 3A,B) to the eNOS antagonist, L-NAME, which should induce vasoconstriction if endogenous NO is contributing to resting diameter, and an even greater vasoconstriction in enlarged collaterals if the high resting diameter was due to excess NO production. As expected, eNOS inhibition vasoconstricted both native (44 ± 1 to 30 ± 1 μm, Figure 3C) and enlarged collaterals (83 ± 3 to 51 ± 2 μm, Figure 3C), but the percent decrease in diameter was not different between groups (−31 ± 1 vs. 38 ± 1%, Figure 3E), indicating that NO production was not driving a sustained “resting” vasodilation in the enlarged collateral. Similar results were observed at day 28, with eNOS inhibition vasoconstricting both native (33 ± 1 to 24 ± 1 μm, Figure 3D) and enlarged collaterals (44 ± 2 to 30 ± 1 μm, Figure 3D); again, the percent decrease in diameter was not different between groups (−27 ± 2 vs. −31 ± 2%, Figure 3F).

**Figure 4:**
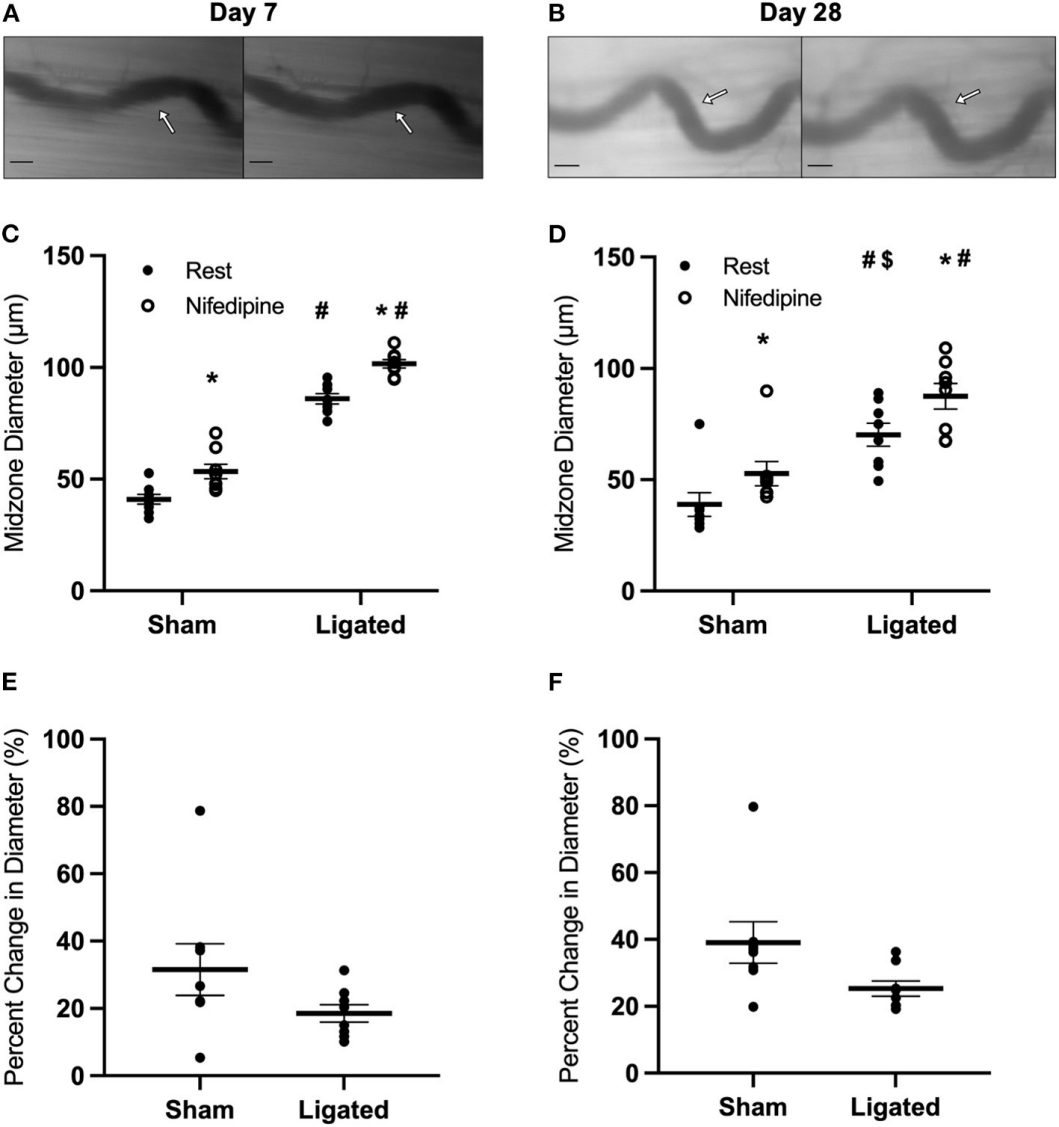
Collateral vessels are not more sensitive to L-type calcium channel inhibition at day 7 and 28. Collateral vessels were observed at the collateral midzone by intravital microscopy with transillumination at 7 days **(A)** or 28 days **(B)** post-femoral artery ligation in the left mouse hindlimb at rest (left) and following exposure to 10^−4^ M of L-type voltage gated channel inhibitor nifedipine (right); arrows indicate the enlarged collateral arteriole and scale bar represents 100 μm. Calcium channel inhibition vasodilates collaterals at both day 7 (**C**, *n* = 8) and day 28 (**D**, *n* = 8). However, there is no difference in the vascular reactivity between the native and enlarged collaterals at both 7 days **(E)** or 28 days **(F)** post-femoral artery ligation. *p < 0.05 vs. resting, #*p* < 0.05 vs. sham, $*p* < 0.05 vs. day 7.

The inability of eNOS inhibition to induce a greater constriction in enlarged collaterals suggests that the higher resting diameter at day 7 following ligation is not due to a maintenance of vasodilation, but rather a loss vascular tone. Resting vascular tone is largely the result of the balance between flow-induced vasodilation signals from the endothelium (e.g., NO) and vasoconstriction induced by myogenic contraction and norepinephrine released from sympathetic neurons at the autonomic neuroeffector junctions (57–59). Therefore, we determined the response of the collateral midzone at 7 or 28 days following ligation (Figures 4A,B) to inhibition of L-type calcium channels, which are involved in myogenic constriction (60, 61); inhibition of these channels should induce vasodilation if they are contributing to resting vascular tone. As expected, at day 7 following ligation, L-type calcium channel inhibition vasodilated the native (41 ± 1 to 53 ± 1 μm, Figure 4C) and enlarged collateral (86 ± 1 to 102 ± 1 μm, Figure 4C), but percent increase in diameter was not different between groups (31 ± 3 vs. 19 ± 1%, Figure 4E). At day 28 following ligation, L-type calcium channel inhibition again vasodilated the native (39 ± 2 to 53 ± 2 μm, Figure 4D) and enlarged collaterals (70 ± 2 to 88 ± 2 μm, Figure 4D), but again the percent increase in diameter was not different between groups (39 ± 2 vs. 25 ± 1%, Figure 4F).

**Figure 5:**
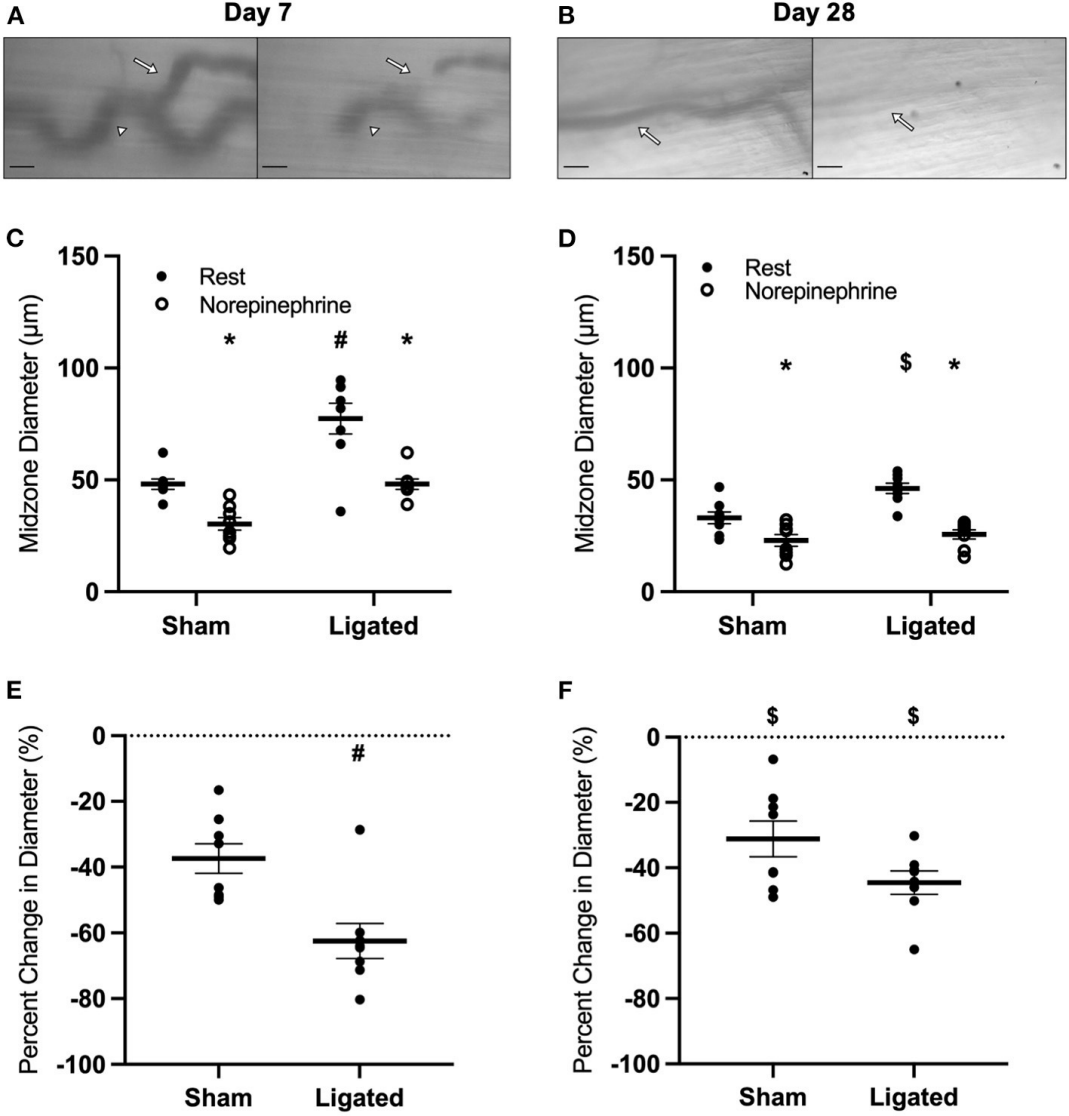
Collateral vessels are normal to hypersensitive to α-adrenergic stimulation at day 7, but not day 28. Collateral vessels were observed at the collateral midzone by intravital microscopy with transillumination at 7 days **(A)** or 28 days **(B)** post-femoral artery ligation in the left mouse hindlimb at rest (left) and following exposure to 10^−4^ M of α-adrenergic agonist norepinephrine (right); regional heterogeneity in the enlarged collateral arteriole to α-adrenergic activation at day 7 is indicated by arrows (responsive, site of measurement) and arrow heads (unresponsive) and scale bar represents 100 μm. While α-adrenergic stimulation vasoconstricts collaterals at both day 7 (**C**, *n* = 8) and day 28 (**D**, *n* = 8), enlarged collaterals appear to be hypersensitive at 7 days **(E)**, but not 28 days post femoral artery ligation **(F)**. *p < 0.05 vs. resting, #*p* < 0.05 vs. sham, $*p* < 0.05 vs. day 7.

The absence of greater vasodilation in response to L-type calcium channel inhibition in the native as compared to the enlarged collaterals suggests that reduced myogenic tone does not explain the higher resting diameter following arteriogenesis. Therefore, we determined the response at the collateral midzone at 7 or 28 days following ligation (Figures 5A,B) to norepinephrine, which contributes to vascular tone when released from vascular sympathetic neurons (62) and should constrict collateral arterioles through α_1_-adrenergic receptors. At day 7 following ligation, norepinephrine vasoconstricted both native (48 ± 3 to 30 ± 3 μm, Figure 5C) and enlarged collaterals (77 ± 2 to 48 ± 1 μm, Figure 5C). Surprisingly, the reactivity was greater in the enlarged collateral than native collateral (−62 ± 2 vs. −37 ± 2%, Figure 5E), indicating a normal-to-hypersensitive response to norepinephrine, when a diminished response was expected. At day 28 following ligation, norepinephrine vasoconstricted both native (33 ± 1 to 23 ± 1 μm, Figure 5D) and enlarged collaterals (46 ± 1, to 26 ± 1 μm, Figure 5D). Unlike day 7, the reactivity was not different between groups (−44 ± 1 vs. −31 ± 2%, Figure 5F).

**Figure 6:**
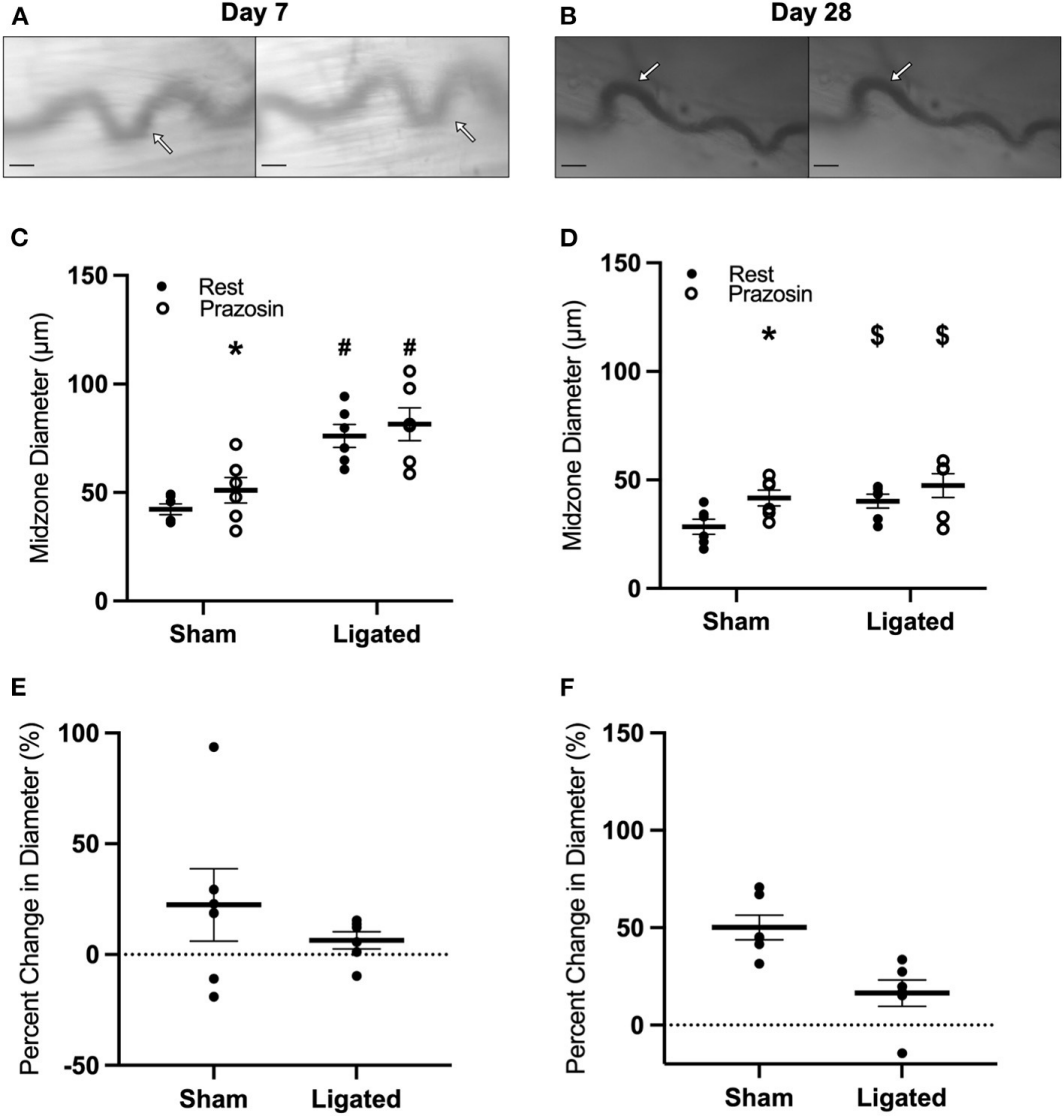
Collateral vessels are insensitive to α-adrenergic inhibition at day 7 and 28. Collateral vessels were observed at the collateral midzone by intravital microscopy with transillumination at 7 days **(A)** or 28 days **(B)** post-femoral artery ligation in the left mouse hindlimb at rest (left) and following exposure to 10^−4^ M of α-1-adrenergic antagonist prazosin (right); arrows indicate the enlarged collateral arteriole and scale bar represents 100 μm. α-adrenergic stimulation vasodilates native, but not enlarged collaterals at both day 7 (**C,E**, *n* = 6) and day 28 (**D,F**, *n* = 6). *p < 0.05 vs. resting, #*p* < 0.05 vs. sham, $*p* < 0.05 vs. day 7.

The loss of vascular tone in the enlarged collateral at day-7 following ligation in the presence of normal-to-hypersensitive response to exogenous norepinephrine suggested that endogenous norepinephrine was not being supplied by muscle sympathetic neurons through the autonomic neuroeffector junctions. Therefore, we determined the response of the collateral midzone at 7 or 28 days following ligation (Figures 6A, 5B) to the α-adrenergic antagonist, prazosin, which should induce vasodilation if secretion of norepinephrine by sympathetic neurons is contributing to resting vascular tone. At day 7 following ligation, prazosin vasodilated the native collateral (42 ± 2 to 51 ± 6 μm, Figure 6C), but had almost no effect on the enlarged collateral (76 ± 5 to 82 ± 8 μm, Figure 6C), translating to a 22 ± 16% increase in the native collateral compared to a 6 ± 4% increase in the enlarged collateral (Figure 6E). Interestingly, once vascular tone had been restored at day 28, prazosin still dilated the native collateral to a greater extent (28 ± 1 to 42 ± 1 μm, Figure 6D) than the enlarged collateral (40 ± 1 to 47 ± 2 μm, Figure 6D), which reflects a 50 ± 3% increase in diameter as compared to a 16 ± 3% increase (Figure 6F). The minimal response to α-adrenergic inhibition at day 7 following ligation is likely not due to the presence of a “maximal” resting diameter in these collaterals, as both muscle contraction (Figure 1C) and L-type calcium channel inhibition (Figure 4C) produce modest increases in enlarged collateral diameter.

**Figure 7:**
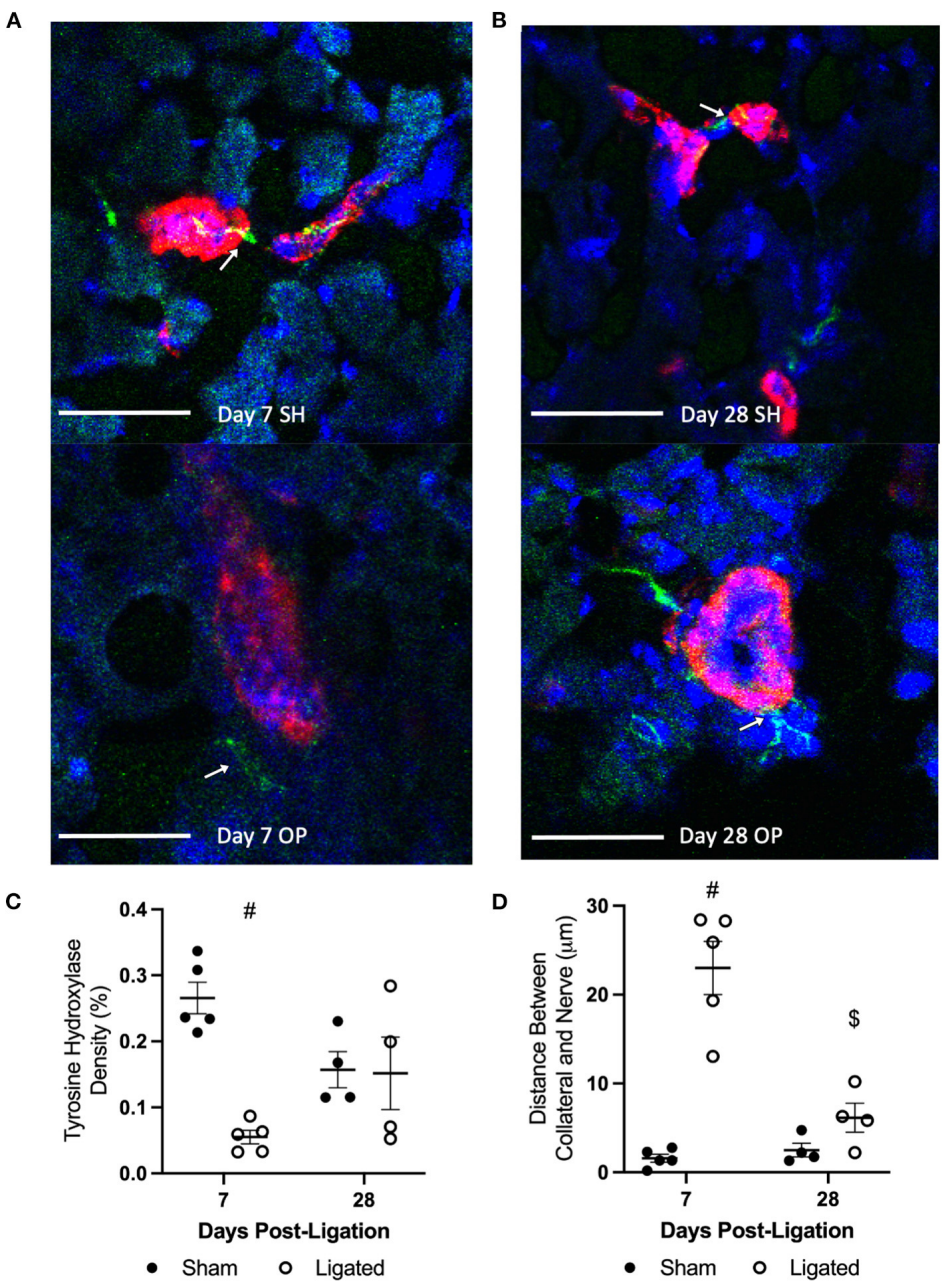
Sympathetic neurons occupy less area and are further from the collateral at day 7. Transverse cross sections of collateral vessel midzone was observed by confocal microscopy at 7 days **(A)** or 28 days **(B)** post-femoral artery ligation in the left mouse hindlimb, after tissue sections of gracilis muscles from ligated (OP, bottom) and sham operated (SH, top) hindlimbs were incubated with anti-α-smooth muscle actin (collateral vessel, red), anti-tyrosine hydroxylase (sympathetic neuron, green, indicated by arrows), and BBI (nuclei, blue); scale bar indicates 50 μm. Sympathetic neurons occupy less area in the peri-collateral region of enlarged collateral at 7 days (**C**, *n* = 5, 3 male and 2 female) but not 28 days (**D**, *n* = 4, 2 male and 2 female). The sympathetic neurons were also further away from the enlarged collateral at 7 days but not 28 day (^#^*p* < 0.05 vs. sham, $*p* < 0.05 vs. day 7).

A loss of vascular tone in the presence of a normal-to-hypersensitive response to NE and an insensitivity of α-adrenergic inhibition indicates that NE is not being released from sympathetic neurons to bind receptors on vascular smooth muscle cells, and suggests that sympathetic neurons may have denervated from the collateral arteriole during enlargement. Therefore, we performed immunofluorescent staining at 7 or 28 days following ligation (Figures 7A,B) for tyrosine hydroxylase, which is the rate-limiting enzyme in the synthesis of catecholamines, such as norepinephrine, from tyrosine amino acids (63), and is expressed by sympathetic neurons in skeletal muscle (64). Consistent with denervation at day 7 following ligation, sympathetic neurons occupied less area in peri-collateral region in the enlarged collateral as compared to native collateral (0.055 ± 0.023 vs. 0.266 ± 0.054%, Figure 7C), and were further from the enlarged collateral than the native collateral (22.99 ± 6.68 vs. 1.58 ± 1.00 μm, Figure 7D). Once vascular tone was restored at day 28, sympathetic neuron area in the peri-collateral region (0.15 ± 0.055 vs. 0.16 ± 0.025%, Figure 7C) and distance from the collateral (6.14 ± 1.64 vs. 2.49 ± 0.765, Figure 7D) were not different between the enlarged and native collaterals, respectively.

**Figure 8:**
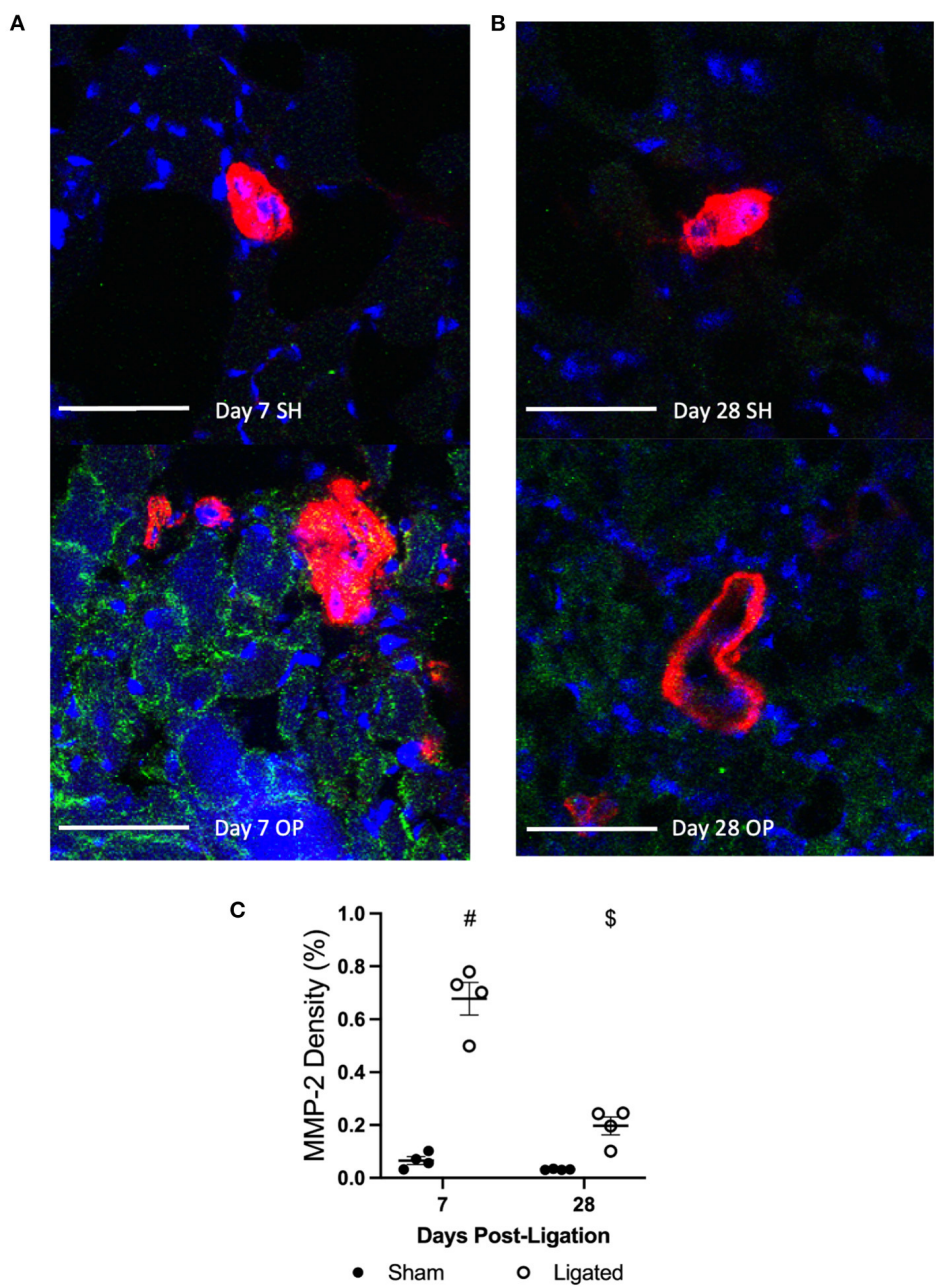
MMP-2 is increased in the pericollateral region at day 7. Transverse cross sections of collateral vessel midzone was observed by confocal microscopy at 7 days **(A)** or 28 days **(B)** post-femoral artery ligation in the left mouse hindlimb after tissue sections of gracilis muscles from ligated (OP, bottom) and sham operated (SH, top) hindlimbs were incubated with anti-α-smooth muscle actin (collateral vessel, red), anti-MMP-2 (indicator of ECM degradation, green), and BBI (nuclei, blue); scale bar indicates 50 μm. MMP-2 enzymes occupy more area in the peri-collateral region of enlarged collateral at 7 days (**C**, *n* = 5, 3 male and 2 female) but not 28 days (*n* = 4, 2 male and 2 female). #*p* < 0.05 vs. sham, $*p* < 0.05 vs. day 7.

To explore a possible mechanism for sympathetic denervation following arteriogenesis, at 7 or 28 days following ligation (Figures 8A,B), we performed immunofluorescent staining for matrix metalloproteinase 2 (MMP2), an extracellular matrix-degrading enzyme. While MMP2 activity is necessary to degrade extracellular matrix and allow vascular cell expansion and collateral enlargement during arteriogenesis (65, 66), it would not be surprising for that activity to separate the relatively weak neuroeffector junctions between vascular smooth muscle cells and sympathetic varicosities. As expected, there is a 10-fold increase in the tissue area positive for MMP2 in the peri-collateral region of the enlarged collateral as compared to the native collateral (0.678 ± 0.12 vs. 0.065 ± 0.029%, Figure 8C). By day 28, once vascular tone is restored and sympathetic neurons are in close proximity to the enlarge collaterals, the peri-collateral area positive for MMP2 is not different between enlarged and native collaterals (0.197 ± 0.0335 vs. 0.032 ± 0.001%, Figure 8C).

## Discussion

Early in the natural history of PAD, patients are commonly asymptomatic, whereas by the end stage of the disease, critical limb ischemia is a leading cause of amputation in the United States (6). Patients with severe cases of PAD have the prognosis of a 20% 5-year survival rate, which is on par with colorectal and pancreatic cancer (7). Additionally, this poor prognosis is not experienced evenly throughout the population, with Black Americans three times more likely to undergo amputation (67). Underlying these poor outcomes is the observation that percutaneous and surgical bypass attempts at revascularization fail to treat almost 50% of patients (6). Clearly, novel treatments for end stage PAD are a major unmet clinical need. Stimulating the enlargement of natural bypass collaterals through arteriogenesis represents an attractive approach for maintaining limb perfusion in PAD. Unfortunately, despite robust success in animal models (12–14) and small clinical trials (15), neither delivery of paracrine factors nor bone marrow-derived cells has improved outcomes in double-blinded, placebo-controlled trials (15–17). We argue that this failure may be due to that fact that enlarged collaterals are not sufficient to restore normal perfusion, but that those collaterals must be able to function as resistance vessels and regulate blood flow through vasodilation and vasoconstriction. Unfortunately, little is known about vascular reactivity during collateral arteriogenesis. Given that vascular proliferation and development of synthetic, non-contractile phenotypes in smooth muscle cells occurs during arteriogenesis, we tested the hypothesis that vasodilation was impaired.

As expected, vascular reactivity was impaired at day 7 and restored by day 28, as measured by percent change in collateral diameter (Figure 1), not because the enlarged collaterals were incapable of vasodilation, as observed in arterialized collateral capillaries (48), but because of a high resting diameter (Figure 1). Giving mice access to a home-cage running wheel decreased resting diameter of the enlarged collaterals (Figure 2) and rescued the impairment in vascular reactivity at day 7 following femoral artery ligation (Figure 2). It is unclear how voluntary exercise accelerates the restoration of vascular tone following collateral arteriogenesis, but it likely involves the production of netrin by smooth muscle cells, which is required for sympathetic innervation of resistance arteries (68). It is possible that increased norepinephrine release during exercise stimulates the vascular smooth muscle to increase the release of netrin. The basis for this suggestion is the observation that sympathetic-derived norepinephrine increases netrin synthesis by macrophages in the context of pulmonary fibrosis (69). It is unclear if vascular smooth muscle cells respond similarly to norepinephrine, but it would not be surprising for a post-synaptic cell to release neurotrophic factors, as occurs at the skeletal myofiber neuromuscular junction (70). Interestingly, vascular tone is also lost following nerve crush injury and muscle injury, and is only restored with neuronal regeneration (71) or muscle regeneration (72), respectively. While we do not expect muscle regeneration in the skeletal muscle containing the enlarged collateral, both arteriogenesis and muscle regeneration involve a robust inflammatory response involving MMP production (73). Regardless of the mechanism, our results indicate that a higher resting diameter is not necessary to match blood flow supply with demand, and in fact, quite the opposite- that the higher resting diameter is a negative side effect of collateral arteriogenesis. Therefore, we determined the mechanism of impaired vascular reactivity during arteriogenesis.

The most likely explanation for a high resting diameter in the enlarged collateral was the NO-dependent shear-induced vasodilation that occurs immediately following ligation (55, 56). However, eNOS inhibition with L-NAME did not cause greater vasoconstriction in the enlarged collateral as compared to the native collateral (Figure 3), so excess NO production was not responsible for the higher resting diameter at day 7 following ligation, as NO production is important for the initial vasodilation response following femoral artery ligation, but not for sustained increases in blood flow or collateral growth (56). If NO is involved in revascularization after stimulating the initial collateral vasodilation, it is likely by supporting angiogenesis in more severe models of hindlimb ischemia (40).

That sustained NO production does not cause the higher resting diameter in the enlarged collateral at day 7 following ligation indicates a loss of vascular tone. The vasoconstricting pathways primarily responsible for maintaining vascular tone are the myogenic response and norepinephrine derived from the sympathetic neuroeffector junctions (32, 57). We expected reduced sensitivity of L-type calcium channels or α-adrenergic receptors, consistent with the synthetic, non-contractile phenotype that is expected in proliferating smooth muscle cells of the enlarging collateral (51, 52, 74), which suppress L-type calcium channels (75) that are involved in the myogenic response (60). The lack of a difference in vasodilation in response to L-type calcium channel inhibition with nifedipine between enlarged and native collaterals (Figure 4) indicates that suppressed L-type calcium channel responses are not the cause of the loss of vascular tone in the enlarged collateral at day 7 following femoral artery ligation and further suggests that the reduction in L-type calcium channel activity may be unique to large vessel injury (76) or could present at earlier time points of collateral arteriogenesis, even though proliferative vascular smooth muscle cells are still observed in the enlarged collateral at day 7 following ligation (77).

With arteriogenesis not affecting NO production nor L-type calcium channel sensitivity, we expected reduced α-adrenergic sensitivity in the enlarged collateral. Surprisingly, norepinephrine produced greater vasoconstriction in the enlarged collateral at day 7 following femoral artery ligation (Figure 5)- exactly opposite of the expected result based on the loss of vascular tone (Figure 1). While the exaggerated response may simply reflect the larger resting diameter, we argue that it could reflect a hypersensitivity due to a loss of gap junctional communication at myoendothelial junctions during the enlargement process. eNOS activation through myoendothelial gap junctions following elevation of intracellular calcium in vascular smooth muscle cells dampens the vasoconstriction response (78). Given the proliferation and reorientation that occurs in the vascular wall during collateral enlargement, it would not be surprising if there were disruptions of the myoendothelial junctions between endothelial cells and vascular smooth muscle cells.

Regardless of the cause of normal-to-hypersensitive response to exogenous norepinephrine, its presence in the context of reduced vascular tone suggested that α-adrenergic receptors were not being activated by endogenous norepinephrine. As expected, the robust vasodilation observed in the native collateral in response to the α-adrenergic antagonist, prazosin, was absent in the enlarged collateral at day 7 following femoral artery ligation (Figure 6). Because enlarged collaterals are capable of modest vasodilation (Figures 1, 4), these pharmacological data suggested that there was anatomical denervation of the sympathetic neuroeffector junctions during collateral enlargement. Tyrosine hydroxylase is required for norepinephrine synthesis, and is commonly used to label sympathetic innervations of resistance vasculature (64). Consistent with the apparent absence of endogenous norepinephrine release, sympathetic neurons occupied less area and were further from the enlarged collateral than the native collateral at day 7, when vascular tone is reduced, but not at day 28, when vascular tone is restored (Figure 7). While a focused investigation into the mechanism of this denervation is outside the scope of this work, the inverse relationship between MMP2 abundance (Figure 8) and sympathetic neuron abundance and proximity (Figure 7) to the collateral suggests that the ECM degradation that is required for collateral arteriogenesis disrupts the sympathetic neuroeffector junctions with vascular smooth muscle cells. Elevated MMP2 activity is a well-characterized aspect of arteriogenesis and our labeling is consistent with previous studies (79).

While the reinnervation of the enlarged collateral temporally correlates to the restoration of vascular tone at day 28, the vascular tone at day 28 still does not appear to be due to norepinephrine release, as α-adrenergic inhibition does not produce vasodilation to a similar extent as the native collateral (Figure 6). This is consistent with a previous work indicating a reduced role for α-adrenergic constriction in restricting collateral-dependent blood flow (33). This may be because the newly matured collateral relies on co-transmitters from sympathetic neurons, such as ATP or neuropeptide Y, both of which induce vasoconstriction (80). While neuropeptide Y was evaluated along with α-adrenergic signaling in restricting collateral-dependent blood flow, it was not evaluated alone (33), so its role is unclear. However, support for an increased relevance of neuropeptide Y in the context of arteriogenesis comes from the observation that neuropeptide Y release is increased and its receptor is upregulated during hindlimb ischemia (81).

There are several functional implications for these novel observations of transient sympathetic denervation & reinnervation and the relationship between these processes and vascular tone. It may be that reinnervation promotes the smooth muscle cell proliferation required for collateral arteriogenesis, as genetic disruption of dopamine β-hydroxylase, which is required for norepinephrine synthesis, reduces capillary arterialization and angiogenesis (82), and α-adrenergic stimulation increases vascular smooth muscle cell proliferation following aortic balloon injury (83). However, it seems more likely that sympathetic reinnervation is more important for maturation of the enlarged collateral, as genetic disruption of dopamine β-hydroxylase does not affect collateral diameter, but reduces medial and advential thickness (82). Indeed, sympathetic neurons have a trophic effect on vascular smooth muscle cells (84), stimulating cerebral arteriogenesis (85) and promoting vascular smooth muscle cell differentiation (86). This vascular smooth muscle cells maturation effect is supported by the observation that genetic disruption of Netrin-1, an axonal guidance cue produced by vascular smooth muscle cells that regulates arterial innervation, leads to defective vasoconstriction (68). While neither collaterals nor skeletal muscle arterioles were evaluated in that report (68), the reduced cold-induced vasoconstriction response observed in skin microvasculature is consistent with the reduced vascular tone we observed in enlarged collaterals (Figure 1).

The apparent requirement of sympathetic innervation for arterial vascular smooth muscle cell maturation raises the question of how a lack of sympathetic reinnervation might impact collateral and resistance vessel function. If vascular smooth muscle cells maturation involves more than the expression of contractile markers, but also includes presumptive reestablishment of myoendothelial junctions, then a lack of reinnervation would be expected to lead to long-term dysfunction in vascular reactivity. Interestingly, patients with PAD exhibit increased vasoconstriction in ischemic skeletal muscle, but not in non-ischemic skeletal muscle (87). In support of this hypothesis is the observation that ischemia-reperfusion injury, a known stimulator of leukocyte recruitment, increases canine arterial contractility to norepinephrine and decreases relaxation to prazosin (88). As mentioned above, while we do not expect ischemia in the enlarging collateral, localized inflammation would be expected in both ischemic zones and enlarging collaterals, suggesting that the impact leukocyte infiltration may upset myoendothelial junctions, which we hypothesize to underlie the apparent hypersensitivity to NE observed following femoral artery ligation (Figure 4).

A potential limitation of this work is the inclusion of adult, as opposed to aged mice, as age is the primary risk factor for PAD (2). However, our research demonstrates how arteriogenesis affects vascular tone and sympathetic innervation when those processes appear to be functioning normally. In the context of advanced age, which impairs arteriogenesis (89), we expect vascular tone and sympathetic denervation/reinnervation to be similarly dysregulated, and the results obtained here provide an excellent foundation to interpret future studies in aged mice. Another potential limitation is the inclusion only of male mice in the vascular pharmacology studies. Female mice were omitted from these studies because differences were not observed between functional vasodilation and arteriogenesis responses in male and female mice, and because vascular reactivity is only expected to be different between male and female mice with hypercholesterolemia (90). Future studies that involve either aged mice or mice with metabolic risk factors should therefore include both male and female mice in all studies.

In summary, arteriogenesis involves a transient loss of vascular tone. The absence of vascular tone correlates with a normal-to-hypersensitive response to norepinephrine and anatomic denervation of sympathetic neuroeffector junctions in the enlarged collateral. Once the enlarged collateral is reinnervated, vascular tone and responses to norepinephrine are normal. When focusing on collateral diameter, it appears as if arteriogenesis is complete when diameter is maximum and vascular cells are no longer proliferating, sometime between 7 (35) and 21 days (54). However, our work demonstrates that once maximum enlargement is achieved, significant maturation processes must still ensue before the enlarged collateral is capable of normal function. This observation seems analogous to early approaches at therapeutic angiogenesis, which focused on increasing capillary number (91, 92), until the realization that a capillary bed stimulated by a single angiogenic factor produced a chaotic and disorganized plexus not unlike the tumor microcirculation (93). Now, it is understood that effective angiogenesis is not just an increased number of capillaries, but their arterial-venous specification, organization into a branching arbor, and arterialization of upstream capillaries into resistance arterioles (94). In an analogous way, we hope that our findings will motivate further investigation into how maturation of the vascular wall following arteriogenesis might affect the functionality and utility of enlarged collaterals. Our findings, coupled with the lack of clinical efficacy in arteriogenesis-stimulating therapies, suggest that further investigation into the natural history of arteriogenesis is necessary to identify therapeutic targets for stimulated enlargement of functional collaterals in patients with PAD.

## Data Availability Statement

The raw data supporting the conclusions of this article will be made available by the authors, without undue reservation.

## Ethics Statement

The animal study was reviewed and approved by California Polytechnic State University IACUC.

## Author Contributions

AS, CH, MC, and TC contributed to the design of the study. AS, CH, and MC conducted the experiments and analyzed the results. All authors wrote sections of the manuscript and contributed to manuscript revisions.

## Funding

This work was supported by CIRM EDUC2-08388.

## Conflict of Interest

The authors declare that the research was conducted in the absence of any commercial or financial relationships that could be construed as a potential conflict of interest.

## Publisher's Note

All claims expressed in this article are solely those of the authors and do not necessarily represent those of their affiliated organizations, or those of the publisher, the editors and the reviewers. Any product that may be evaluated in this article, or claim that may be made by its manufacturer, is not guaranteed or endorsed by the publisher.
